# Phenformin, But Not Metformin, Delays Development of T Cell Acute Lymphoblastic Leukemia/Lymphoma via Cell-Autonomous AMPK Activation

**DOI:** 10.1016/j.celrep.2019.03.067

**Published:** 2019-04-16

**Authors:** Diana Vara-Ciruelos, Madhumita Dandapani, Fiona M. Russell, Katarzyna M. Grzes, Abdelmadjid Atrih, Marc Foretz, Benoit Viollet, Douglas J. Lamont, Doreen A. Cantrell, D. Grahame Hardie

**Affiliations:** 1 Division of Cell Signalling & Immunology, College of Life Sciences, University of Dundee, Scotland, UK; 2 Fingerprints Proteomics Facility, College of Life Sciences, University of Dundee, Scotland, UK; 3 Inserm U1016, Institut Cochin, Paris, France; 4 CNRS UMR8104, Paris, France; 5 Université Paris Descartes, Sorbonne Paris cité, Paris, France

**Keywords:** AMP-activated protein kinase, AMPK, biguanides, metformin, phenformin, T-ALL, T cell acute lymphoblastic leukemia/lymphoma

## Abstract

AMPK acts downstream of the tumor suppressor LKB1, yet its role in cancer has been controversial. AMPK is activated by biguanides, such as metformin and phenformin, and metformin use in diabetics has been associated with reduced cancer risk. However, whether this is mediated by cell-autonomous AMPK activation within tumor progenitor cells has been unclear. We report that T-cell-specific loss of AMPK-α1 caused accelerated growth of T cell acute lymphoblastic leukemia/lymphoma (T-ALL) induced by PTEN loss in thymic T cell progenitors. Oral administration of phenformin, but not metformin, delayed onset and growth of lymphomas, but only when T cells expressed AMPK-α1. This differential effect of biguanides correlated with detection of phenformin, but not metformin, in thymus. Phenformin also enhanced apoptosis in T-ALL cells both *in vivo* and *in vitro*. Thus, AMPK-α1 can be a cell-autonomous tumor suppressor in the context of T-ALL, and phenformin may have potential for the prevention of some cancers.

## Highlights

• AMPK knockout in T cells accelerates acute lymphoblastic leukemia/lymphoma (T-ALL)
• Oral treatment with phenformin delays T-ALL development in AMPK-dependent manner
• Oral metformin has no effect, likely because of the lack of cellular uptake by thymocytes
• Phenformin lowers glycolysis and oxidative metabolism, reducing T-ALL cell viability

## Graphical Abstract

**Figure:**
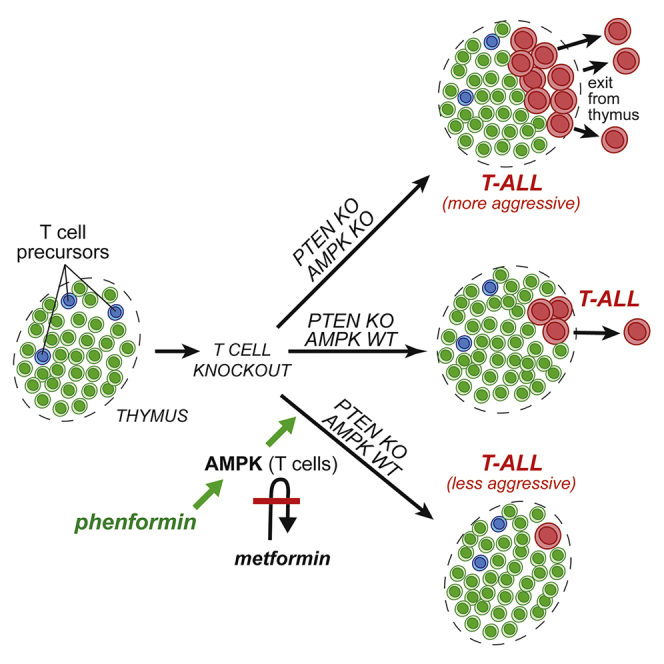

## Teaser

The roles of AMPK in cancer and of biguanides in its prevention or treatment are controversial. Vara-Ciruelos et al. now report that genetic loss of AMPK in T cells accelerates T cell acute lymphoblastic leukemia/lymphoma, whereas the biguanide phenformin, but not metformin, protects against its development in a cell-autonomous, AMPK-dependent manner.

## Introduction

The biguanides metformin and phenformin are derived from a natural product of the plant *Galega officinalis*, used as an herbal medicine in medieval Europe. Although introduced for treatment of type 2 diabetes in the 1950s, their molecular mechanism only began to emerge much later with findings that they inhibited complex I of the respiratory chain, thus repressing mitochondrial ATP synthesis (Bridges et al., 2014, El-Mir et al., 2000, Owen et al., 2000). This increases cellular AMP:ATP ratios, leading to activation of AMP-activated protein kinase (AMPK) (Hawley et al., 2010, Zhou et al., 2001). Although the ability of metformin to acutely inhibit hepatic glucose production is independent of AMPK (Foretz et al., 2010), its longer-term ability to reverse insulin resistance is dependent upon phosphorylation of acetyl-coenzyme A (CoA) carboxylase by AMPK (Fullerton et al., 2013).

Metformin is a cation with low membrane permeability and is not taken up significantly by cells lacking transporters of the organic cation transporter family (e.g., organic cation transporter [OCT]1), although phenformin enters cells lacking such transporters (Hawley et al., 2010). Once taken up, biguanides concentrate in the cytoplasm and then in mitochondria, driven by their positive charge and the membrane potentials across the respective membranes (Owen et al., 2000).

AMPK is a sensor of cellular energy status; once activated, it switches on catabolic pathways generating ATP and switches off most biosynthetic pathways and progress through the cell cycle (Ross et al., 2016). AMPK is only active after phosphorylation by the tumor-suppressor kinase LKB1, originally identified as the gene mutated in an inherited susceptibility to cancer termed Peutz-Jeghers syndrome (Alessi et al., 2006). LKB1 is also frequently mutated in spontaneous cancers, particularly lung adenocarcinomas (Ross et al., 2016). This link between AMPK and a tumor suppressor led to studies showing that diabetics taking metformin had a lower cancer incidence than those on other medications (Evans et al., 2005). Although supported by several subsequent studies, the validity of that association has been challenged (Suissa and Azoulay, 2014), and one controlled trial of metformin in pancreatic cancer yielded negative results (Kordes et al., 2015).

A key question concerns the extent to which the tumor-suppressor functions of LKB1 are mediated by AMPK. One study used mice lacking the *Prkaa1* gene encoding AMPK-α1, the sole catalytic subunit isoform expressed in lymphocytes. Consistent with the idea that AMPK is a tumor suppressor, gene deletion caused acceleration of lymphomas triggered by c-Myc expression in B cells (Faubert et al., 2013). A drawback with that model was that AMPK-α1 was deleted globally, not just in B cells. Other studies suggested that the presence of either LKB1 (Algire et al., 2011, Shackelford et al., 2013) or AMPK (Jeon et al., 2012, Kishton et al., 2016) increased survival of tumor cells during nutrient or oxygen deprivation or oxidative stress, thus exerting tumor-promoting effects. Moreover, analysis of human cancer genome databases showed that the genes encoding AMPK-α1 and AMPK-β2 are frequently amplified, consistent with roles in promoting tumorigenesis (Ross et al., 2016).

To address the role of AMPK in T cell acute lymphoblastic leukemia/lymphoma (T-ALL), we used a mouse model in which the tumor suppressor gene *Pten* was deleted in T cell progenitors (Hagenbeek and Spits, 2008), and generated lines with or without additional T-cell-specific knockout of AMPK-α1. We also tested the effects of biguanides in those mice.

## Results

### Deletion of AMPK-α1 Accelerates Development of T-ALL Induced by PTEN Loss

We generated mice with T-cell-specific deletion of PTEN and/or AMPK-α1 by crossing *Pten*^*fl/fl*^ and *Prkaa1*^*fl/fl*^ mice with *Lck-Cre* transgenic mice. Because α1 is the only catalytic subunit isoform expressed in T cells, with no α2 expression, even when α1 is knocked out (Rolf et al., 2013), we refer to these as tPTEN^−/−^ tAMPK^+/+^ (AMPK wild type [WT]), tPTEN^−/−^ tAMPK^+/−^ (heterozygous deletion), or tPTEN^−/−^ tAMPK^−/−^ (homozygous deletion). Mice were monitored daily until showing malaise, when thymus, lymph nodes, and spleen were inspected for lymphoma. Tumors were found either in the thymus only or in thymus and other lymphoid organs.

**Figure 1:**
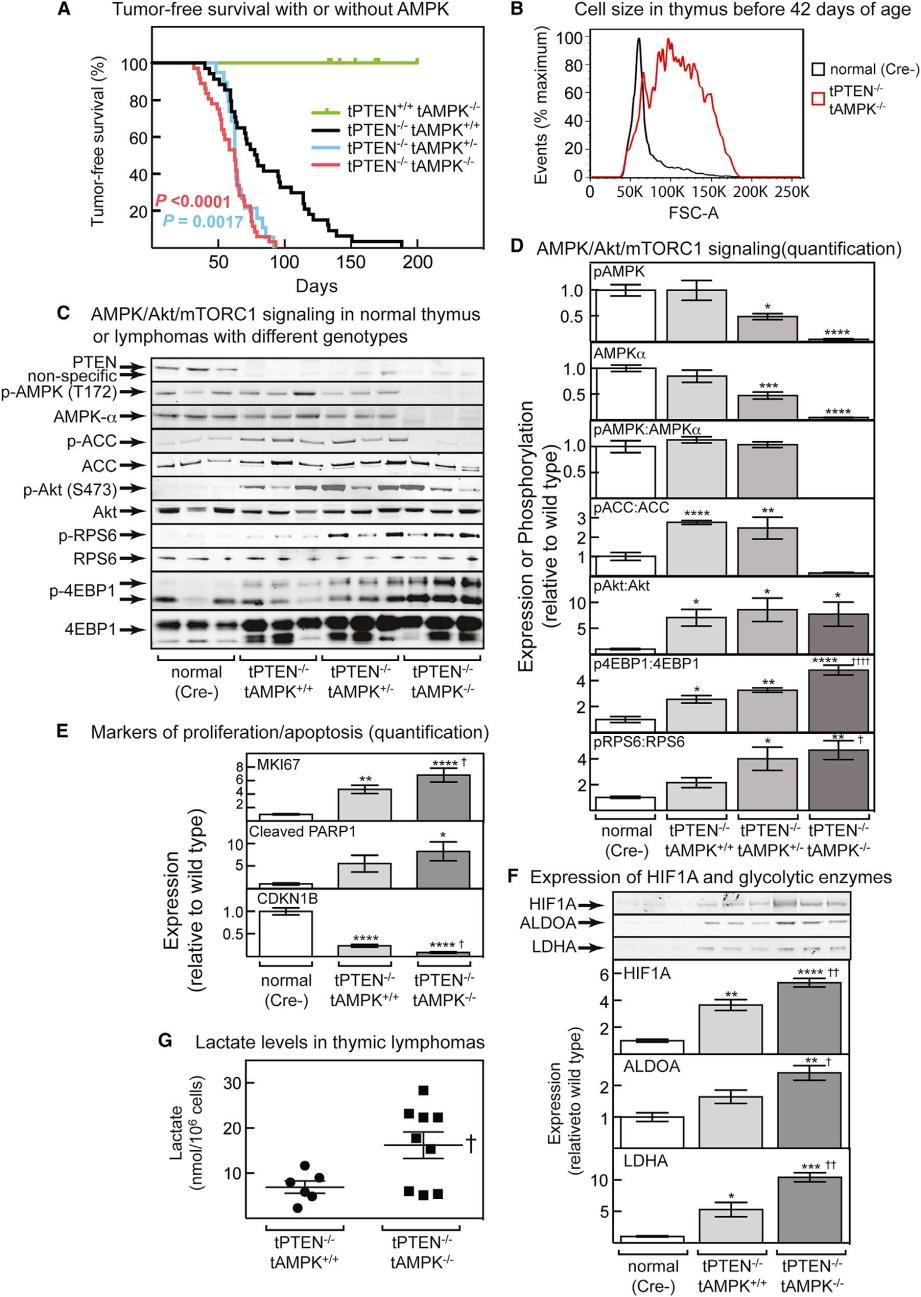
T-Cell-Specific Loss of AMPK Accelerates Development of T-ALL and Causes mTORC1 Hyperactivation (A) Tumor-free survival curves for mice with T cells of the four different genotypes. p values (log-rank, Mantel Cox) for survival curves that are significantly different from those of the tPTEN^−/−^ tAMPK^+/+^ mice are shown. (B) Distribution of cell sizes, estimated by forward scatter in flow cytometry, of thymocytes from mice of two different genotypes at 37–42 days of age. The population of large cells in the tPTEN^−/−^ tAMPK^−/−^ sample indicates incipient lymphoma. (C) Signaling via AMPK, Akt, and mTORC1 in lymphomas from three mice of each genotype. (D) Quantification of blots from all mice analyzed; for pAMPK, AMPKα, pAMPK:AMPKα, pACC:ACC, and pAkt:Akt (Ser473), n = 8; for pRPS6:RPS6 and pEBP1:4EBP1, n = 6 to 10. (E) Quantification of blots (see Figure S1 for representative blots) analyzing expression of markers of cell proliferation and apoptosis in normal thymus or lymphomas of the indicated genotypes (n = 8–10). (F) Expression of HIF1A and the glycolytic enzymes aldolase A (ALDOA) and lactate dehydrogenase A (LDHA). (Top) Western blots from three mice. (Bottom) Quantification for all samples analyzed (HIF1A and LDHA, n = 3–5; ALDOA, n = 6–10. (G) Lactate levels in lymphoma-bearing thymus of the indicated genotypes. (A–G) Mean values ± SEM are shown; those significantly different from the Cre- control by one-way ANOVA are indicated by asterisks, and those significantly different from tPTEN^−/−^ tAMPK^+/+^ mice are indicated by daggers (†).

As shown previously (Hagenbeek and Spits, 2008), mice with T-cell-specific PTEN loss began to develop lymphomas at about 50 days of age, and almost all had developed tumors by 150 days (Figure 1A). Mice with T-cell-specific loss of AMPK-α1 displayed no tumors up to 200 days. However, mice with loss of both PTEN and AMPK-α1 developed tumors earlier, and subsequent tumor-free survival was greatly reduced (Figure 1A). Surprisingly, mice with a single *Prkaa1* allele behaved similarly to those that had lost both alleles, except that the tumors did not arise earlier. Median tumor-free survival was 94 days for PTEN-null mice with WT AMPK, and 75 days for those with either heterozygous or homozygous deletion of AMPK; the hazard ratios (Mantel-Haenszel), were 3.2 for heterozygous and 3.6 for homozygous AMPK deletion.

To confirm that tumors arose earlier with homozygous AMPK deletion, we examined thymus of some mice at 29–42 days of age, before external signs of disease were evident. Incipient lymphoma could be recognized by the presence of a population of large cells detectable by flow cytometry; Figure 1B illustrates one example of this in a tPTEN^−/−^tAMPK^−/−^ mouse compared with a normal Cre recombinase-negative (Cre-) control. The presence of large cells was much less frequent when PTEN alone was deleted (2 of 14 cases, 16%) or when PTEN plus one AMPK allele was deleted (1 of 6, 16%) than when PTEN and both alleles of AMPK were deleted (13 of 22, 60%). The difference between the PTEN-null and PTEN/AMPK-null mice was significant (one-tailed Fisher’s exact test, p = 0.009).

### Analysis of Protein Expression and Cell Signaling in T-ALL Cells

We next monitored expression and/or phosphorylation of cell-signaling markers in lymphomas, compared with normal thymocytes from age-matched controls. Figure 1C shows thymus blots from three representatives of each genotype, whereas Figure 1D shows quantification of blots from all samples analyzed. As expected, the levels of Thr172 phosphorylation (pAMPK) and AMPK-α expression were reduced by around 50% in the AMPK heterozygote (although the pAMPK:AMPK-α ratio did not change), and the AMPK-α and p-Thr172 signals were almost eliminated by homozygous AMPK deletion (Figures 1C and 1D). Interestingly, PTEN deletion caused a significant increase in the phosphorylation of the AMPK target acetyl CoA carboxylase (ACC); similar results were obtained with WT AMPK or heterozygotes, although ACC phosphorylation was almost eliminated by homozygous deletion. As expected, PTEN deletion increased phosphorylation of Akt at Ser473 in tumors, but this did not increase further on AMPK deletion. However, phosphorylation of two downstream targets of mTORC1, 4EBP1 and RPS6, did increase further on AMPK deletion, showing that deleting PTEN and AMPK had additive effects to promote mTORC1 activity. PTEN-null lymphomas, compared with normal thymocytes, also expressed high levels of markers of proliferation (MKI67) and apoptosis (cleaved PARP1), and low levels of the G1 cyclin-dependent kinase inhibitor CDKN1B; these changes were further enhanced when AMPK was knocked out (Figure 1E; representative blots shown in Figure S1).

A key protein whose expression is controlled by mTORC1 signaling in T cells is hypoxia-inducible factor-1α (HIF1A) (Finlay et al., 2012). PTEN-null thymic tumors expressed high levels of HIF1A, as shown previously (Grzes et al., 2017), and that increased further on AMPK deletion (Figure 1F). Similar changes of expression were observed for two transcriptional targets of HIF1A, the glycolytic enzymes aldolase-A (ALDOA) and lactate dehydrogenase-A (LDHA). Interestingly, lactate content was also significantly increased in thymic lymphomas from mice with deletion of both PTEN and AMPK compared with PTEN alone (Figure 1G).

### Deletion of AMPK Did Not Affect T Cell Development

We wondered whether tumorigenesis might be more rapid after AMPK deletion because that affected T cell development, perhaps by increasing the pool of PTEN-null T cell progenitors that generate the tumors. However, flow cytometric analysis revealed no difference in number or frequency of different thymocyte sub-populations in tPTEN^−/−^ tAMPK^+/+^ or tPTEN^−/−^ tAMPK^−/−^ mice analyzed at 37–42 days (Figure S2). Similarly, no differences were detected in cells from spleen or lymph nodes (data not shown).

### Effect of Metformin and Phenformin on Tumor-free Survival *In Vivo*

The results above revealed that the absence of AMPK in T cell progenitors accelerates the growth of lymphomas induced by PTEN loss. We also tested the converse, i.e., whether AMPK activation *in vivo* with a biguanide would exert an anti-tumor effect. We first examined effects of delivery of metformin in drinking water (calculated to deliver 400 mg/kg), starting at 30 days of age in tPTEN^−/−^ tAMPK^+/+^ and tPTEN^−/−^ tAMPK^−/−^ mice. That dose is greater than that used in other mouse studies (250 mg/kg), in which plasma metformin concentrations reached 3–12 μM, similar to those achieved in humans on the standard dose of ≈30 mg/kg (Chandel et al., 2016). Despite that relatively high dose, there were no significant changes in survival caused by metformin, irrespective of whether T cells expressed AMPK (Figure S3A). A likely explanation was that metformin cannot activate AMPK in thymus because of the lack of suitable transporters for cell uptake. Consistent with that, metformin (although detectable in plasma and liver) could not be detected in the thymus after a single administration by oral gavage (Figure S3B). In addition, metformin treatment did not increase phosphorylation of AMPK or ACC in lymphomas from tPTEN^−/−^ tAMPK^+/+^ mice (Figures S3C and S3D). Attempts to detect OCT family members by western blotting were also unsuccessful, in line with proteomic analyses of tPTEN^−/−^ T-ALL cells, in which no expression of any member of the OCT family was detected (data deposited with ProteomeXchange Consortium: PXD006209 [Grzes et al., 2017]).

**Figure 2:**
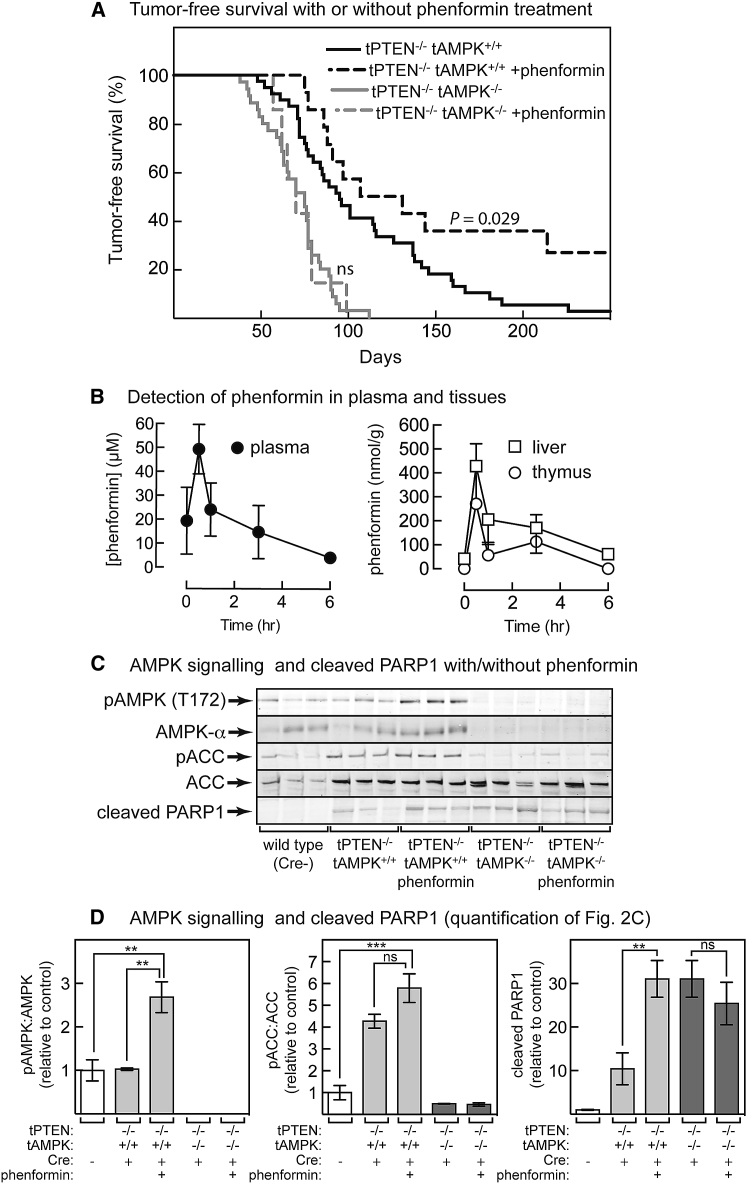
Effect of Phenformin on Tumor-free Survival and Signaling in the Thymus *In Vivo* (A) Effect of phenformin (300 mg/kg) on tumor-free survival in tPTEN^−/−^ tAMPK^+/+^ or tPTEN^−/−^ tAMPK^−/−^ mice. The effect was significant as shown (log-rank, Mantel-Cox) only in tumors with wild-type AMPK-α1. Median survival was as follows: tPTEN^−/−^ tAMPK^−/−^, 75 days; tPTEN^−/−^ tAMPK^−/−^ plus phenformin, 70 days; tPTEN^−/−^ tAMPK^+/+^, 95 days; tPTEN^−/−^ tAMPK^+/+^ plus phenformin, 119 days. (B) Effects of time after oral gavage with phenformin on recovery (by LC:MS) in plasma (left) and liver and thymus (right). Results are means ± SEM (n = 3) in wild-type mice of 29–42 days of age. (C) Expression and phosphorylation of AMPK and ACC and cleaved PARP1 in thymic lymphomas of mice treated with or without phenformin (n = 3), sampled when animals became unwell, or thymus of age-matched controls. (D) Quantification of blots in (C); results are means ± SEM. Results significantly different by one-way ANOVA are indicated by asterisks; ns, not significant.

Unlike metformin, phenformin significantly increased tumor-free survival, but that was dependent on cell-autonomous expression of AMPK because no effect was seen in tPTEN^−/−^ tAMPK^−/−^ mice (Figure 2A). Phenformin was also detected after oral gavage in thymus as well as plasma and liver (Figure 2B), correlating with enhanced phosphorylation of Thr172 on AMPK in lymphomas expressing AMPK-α1 (Figures 2C and 2D). Phosphorylation of ACC also appeared to increase, although that was not statistically significant, perhaps because phosphorylation was already high in mice with PTEN deletion. Phenformin treatment also increased apoptosis in the lymphoma cells as shown by a large increase in cleaved PARP1, although only when AMPK was present (Figures 2C and 2D).

### Effect of Biguanides on Tumor Cell Viability *In Vitro*

**Figure 3:**
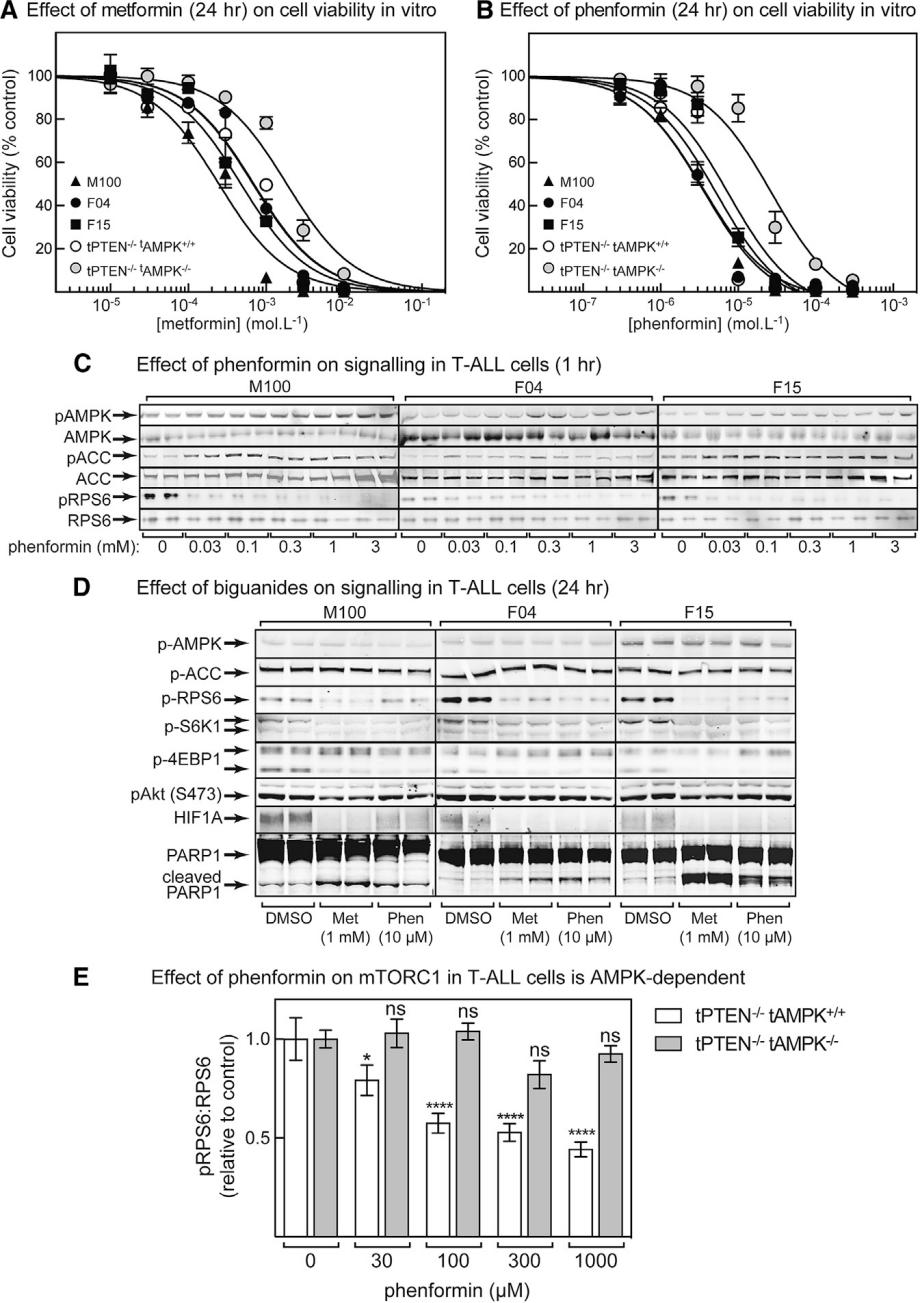
Cell Viability and Expression of Markers during Treatment of Cells with Biguanides (A) Cell viability by flow cytometry (using DAPI to label dead cells) for three established T-ALL cell lines (M100, F04, and F15) and for primary lymphoma cells derived from mice with T-cell-specific deletion of PTEN and/or AMPK after incubation for 24 h in the indicated concentrations of metformin. Results (means ± SEM, n = 8) were fitted as described in the STAR Methods. (B) As shown in (A) but with phenformin. (C) Phosphorylation of AMPK, ACC and RPS6 after 1 hr treatment with different concentrations of phenformin (n = 2). (D) Western blotting of various markers in tPTEN^−/−^ T-ALL cell lines treated for 24 h with 1 mM metformin or 10 μM phenformin (n = 2). (E) Effect of incubation with phenformin (1 h) on mTORC1 signaling (pRPS6:RPS6 ratio) in T-ALL cells with or without AMPK. Results are means ± SEM (n = 4); significance of differences from vehicle control are indicated by asterisks (ns, not significant).

To investigate how phenformin protected against tumorigenesis in an AMPK-dependent manner, we tested effects of biguanides on tumor cell viability *in vitro*, both with primary PTEN-null T-ALL cells from this project and with three previously established PTEN-null T-ALL lines (M100, F04, and F15). Metformin reduced the viability of all three established lines, although only at high concentrations (Figure 3A); the half-maximal inhibitory concentration (IC_50_) values ranged from 200 to 700 μM (peak plasma concentration observed *in vivo* was 200 μM; Figure S3B). The estimated IC_50_ value (±SEM) for primary lymphoma cells lacking PTEN was similar at 700 ± 100 μM, whereas, in cells lacking both PTEN and AMPK, it was higher at 1.8 ± 0.2 mM.

Phenformin was much more potent (Figure 3B; note different scale on horizontal axis from Figure 3A); for the three established cell lines, the IC_50_ values ranged from 3 to 7 μM (well below the estimated peak plasma phenformin concentration of 50 μM; Figure 2B). For primary lymphoma cells lacking PTEN, the IC_50_ value was similar (5 ± 1 μM), whereas for cells lacking both PTEN and AMPK, the IC_50_ was higher (27 ± 5 μM). Figure 3C shows that similar phenformin concentrations increased phosphorylation of AMPK and ACC and decreased phosphorylation of RPS6 after 1 h, although the effect on AMPK was small.

Figure 3D shows western blots of the tPTEN^−/−^ T-ALL cell lines treated with metformin (1 mM) or phenformin (10 μM) for 24 h. Although phosphorylation of AMPK and ACC had returned to baseline by that time, other downstream effects were more evident. At that point, both biguanides still inhibited mTORC1, as judged by reduced phosphorylation of RPS6, S6K1, and 4EBP1 and reduced HIF1A expression, and both also increased apoptosis, as assessed by PARP1 cleavage. Figure 3E shows the effects of phenformin on mTORC1 function in primary lymphoma cells, revealing reduced RPS6 phosphorylation that was AMPK dependent because it was absent in AMPK knockout cells.

### Effects of Phenformin and Rapamycin on T-ALL Cells *In Vitro*

**Figure 4:**
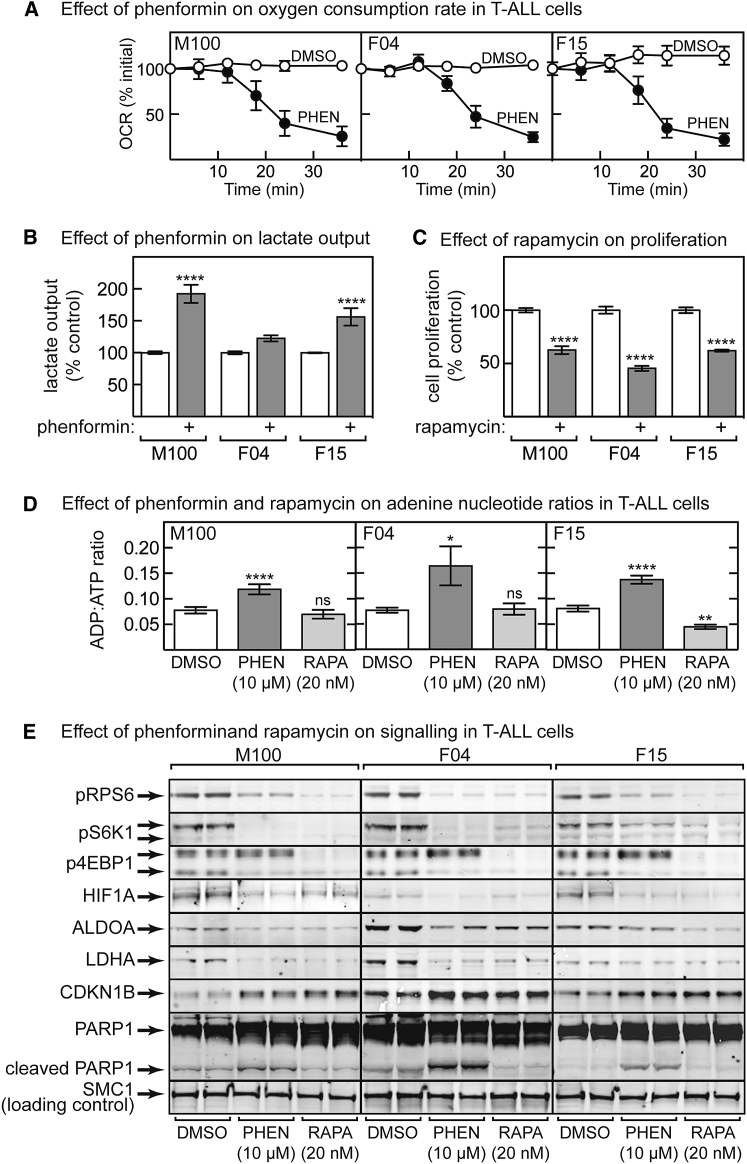
Effects of Phenformin and Rapamycin in Established tPTEN^−/−^ T-ALL Cell Lines (A) Effect of time of incubation with 10 μM phenformin on oxygen consumption rate (OCR); results are means ± SEM (n = 3). (B) Effect of phenformin treatment (10 μM) for 48 h on lactate output; results are means ± SEM (n = 4–6). (C) Effect of rapamycin (20 nM) on cell proliferation. Cells were grown for 48 h before 3-(4,5-dimethylthiazol-2-yl)-2,5-diphenyltetrazolium bromide (MTT) assay; results are percentage of the control ± SEM (n = 6). (D) Cellular ADP:ATP ratios in cell lines treated for 48 h with phenformin or rapamycin. Results are means ± SEM (n = 6 for M100 cells; n = 8 for F01/F15 cells). (E) Analysis by western blot of cell lines (n = 2) treated for 48 h with vehicle (DMSO), phenformin (10 μM), or rapamycin (20 nM). In (B)–(D), results significantly different from DMSO controls are indicated by asterisks.

To gain more clues as to how phenformin protected against lymphoma, we further analyzed the three established T-ALL lines *in vitro*. As expected for a respiratory-chain inhibitor, phenformin caused a progressive decrease in oxygen uptake (Figure 4A). This was associated with increased lactate output, although the effect was not significant in F04 cells (Figure 4B). As expected, the mTORC1 inhibitor rapamycin decreased cell proliferation (Figure 4C). Phenformin caused increases in the cellular ADP:ATP ratio in all three lines, whereas rapamycin either had no effect or, in F15 cells, even decreased it (Figure 4D). Both phenformin and rapamycin inhibited the mTORC1 pathway, as assessed by reduced phosphorylation of RPS6, S6K1, and 4EBP1 and reduced expression of HIF1A and the glycolytic enzymes ALDOA and LDHA. Both also increased expression of the G1 cyclin-dependent kinase inhibitor CDKN1B, consistent with inhibition of cell proliferation. However, a striking difference was that increased apoptosis, as assessed by PARP1 cleavage, was only observed in cells treated with phenformin and not with rapamycin (Figure 4E).

## Discussion

Although loss of AMPK-α1 alone did not cause lymphomas, it accelerated development of lymphomas triggered by PTEN loss. Because loss of AMPK-α1 was T cell specific, we can be certain that this was a cell-autonomous effect, unlike a previous study (Faubert et al., 2013) that used a B cell lymphoma model. Surprisingly, the effects of heterozygous and homozygous deletion of AMPK-α1 on tumor-free survival were similar, showing that the tumor-suppressor effect of AMPK displays haploinsufficiency.

In this mouse model, AMPK, therefore, acts as a tumor suppressor. Rathmell’s group (Kishton et al., 2016) used a different model of T-ALL in which oncogenic NOTCH1 was expressed in murine hematopoietic stem cells that carried a floxed AMPK-α1 gene and Cre recombinase driven by a tamoxifen-inducible promoter. The cells were multiplied in irradiated mice and, then, injected into irradiated, secondary recipients. By treating the latter with tamoxifen, AMPK-α1 could be acutely deleted in the T-ALL cells. In this model, knocking out AMPK-α1 reduced recovery of T-ALL cells in spleen, lymph nodes, and bone marrow and enhanced mouse survival (Kishton et al., 2016). Thus, although the presence of two WT AMPK-α1 alleles protected against T-ALL development in our model, once tumor cells had developed, the presence of AMPK-α1 enhanced T-ALL cell viability and reduced mouse survival. Thus, although AMPK acts as a tumor suppressor during T-ALL development, once tumors have arisen, it switches to being a tumor promoter.

Because Akt activation and AMPK inhibition activate mTORC1 by distinct mechanisms, it is not surprising that deleting both PTEN and AMPK in T cells caused mTORC1 hyper-activation in the resultant lymphomas. This was associated with increased expression of HIF1A and two of its downstream targets, the glycolytic enzymes ALDOA and LDHA. Consistent with increased activation of glycolysis on AMPK deletion, lactate levels were higher in the lymphomas with both PTEN and AMPK deleted.

Because AMPK loss accelerated lymphoma development, we tested whether the converse was true, i.e., whether pharmacological activation of AMPK would provide protection. Intriguingly, although metformin (even at high doses) had no effect, phenformin significantly delayed tumorigenesis, although only when AMPK-α1 was present in T cells. Thus, the protective effect of phenformin in T-ALL is both AMPK dependent and cell autonomous. The failure of metformin to provide protection correlated with its inability to activate AMPK in thymic lymphomas, most likely because of the lack of expression of transporters, such as OCT1; expression of OCT1 family members was not detected in a previous proteomic analysis of PTEN-null T-ALL cells (Grzes et al., 2017). Moreover, although we could detect metformin by liquid chromatography-mass spectrometry (LC-MS) in liver and plasma, it was undetectable in thymic tumors from the same animals. By contrast, we could detect phenformin in liver, plasma, and thymic tumors. Thr172 phosphorylation on AMPK in thymus was also enhanced by phenformin, but not metformin. Unlike metformin, the more-hydrophobic phenformin is readily taken up by cells lacking OCT1 (Hawley et al., 2010). Indeed, phenformin was around 100-fold more potent than metformin in reducing the viability of T-ALL cells *in vitro*, correlating with greater potency in phosphorylation of AMPK and ACC. For phenformin, unlike metformin, the IC_50_ for reduced viability *in vitro* was much lower than the peak plasma concentrations observed *in vivo*.

In three established T-ALL cell lines, both phenformin and rapamycin inhibited mTORC1; reduced expression of HIF1A, ALDOA, and LDHA; and increased expression of the cell cycle inhibitor CDKN1B. However, a striking difference was that only phenformin triggered apoptosis, as assessed by PARP1 cleavage. Phenformin also triggered apoptosis *in vivo*, although only when T-ALL cells expressed AMPK (Figures 2C and 2D). A potential explanation for that difference is that, although phenformin and rapamycin have similar effects to downregulate glycolysis by inhibiting mTORC1 and hence HIF1A expression, phenformin also inhibits oxidative metabolism. Thus, cells treated with rapamycin can switch to oxidative metabolism to generate ATP, but cells treated with phenformin cannot and undergo apoptosis instead. Consistent with this, phenformin, unlike rapamycin, increased the cellular ADP:ATP ratios in all three T-ALL cell lines and increased apoptosis in an AMPK-dependent manner in lymphoma cells *in vivo*.

If these findings are relevant in humans, biguanides may be useful in preventing certain types of cancer in which AMPK acts as a tumor suppressor, especially those displaying hyper-activated mTORC1. However, many tumor cells may not express transporters required for metformin uptake, and in such cases, the more cell-permeable biguanide phenformin should be considered instead. Although it was withdrawn for diabetes treatment because of its association with lactic acidosis, that side effect was quite rare (≈64 cases per 100,000 patient-years [Crofford, 1995]). Although unacceptable for treatment of diabetes, this risk may be tolerable in cancer.

## STAR★Methods

### Key Resources Table

**Table:** 

REAGENT or RESOURCE	SOURCE	IDENTIFIER
**Antibodies**		

PTEN	Santa Cruz	Cat# Sc7974; RRID:AB_628187
pT172 (AMPK-α)	Cell Signaling Technology	Cat# 2531; RRID: AB_330330
AMPK, pan-α (for western blots)	Cell Signaling Technology	Cat# 2532; RRID: AB_330331
AMPK-α1 (for immunoprecipitation)	(Woods et al., 1996)	N/A
AMPK-α2 (for immunoprecipitation)	(Woods et al., 1996)	N/A
pACC1/pACC2 (S79/S212)	Cell Signaling Technology	Cat# 11818; RRID: AB_2687505
ACC	Cell Signaling Technology	Cat# 2280; RRID: AB_10694695
pAkt (T308)	Cell Signaling Technology	Cat# 9275; RRID:AB_329828
pAkt (S473)	Cell Signaling Technology	Cat# 4060; RRID:AB_2315049
Akt	Cell Signaling Technology	Cat# 2920; RRID:AB_1147620
p-RPS6 (S235/S236)	Cell Signaling Technology	Cat# 2211; RRID:AB_331679
RPS6	Cell Signaling Technology	Cat# 2317; RRID:AB_2238583
p4EBP1 (T37/T46)	Cell Signaling Technology	Cat# 2855; RRID:AB_560835
4EBP1	Cell Signaling Technology	Cat# 9644; RRID:AB_2097841
pS6K1 (T389)	Cell Signaling Technology	Cat# 9234; RRID:AB_2269803
S6K1	Cell Signaling Technology	Cat# 2708; RRID:AB_390722
MKI67	Abcam	Cat# ab15580; RRID:AB_443209
CDKN1B	Cell Signaling Technology	Cat# 3688; RRID:AB_2077836
PARP1	Cell Signaling Technology	Cat# 9532; RRID:AB_659884
HIF1A	Cell Signaling Technology	Cat# 3716; RRID:AB_2116962
ALDOA	Cell Signaling Technology	Cat# 8060
LDHA	Cell Signaling Technology	Cat# 3582; RRID:AB_2066887
SMC1	Bethyl	Cat# A300-055ª; RRID:AB_2192467
CD4 PE-Cy7	BD Biosciences	Cat# 552775; RRID: AB_394461
CD8a V450	BD Biosciences	Cat# 560469; RRID:AB_1645281
CD71-PE	BD Biosciences	Cat# 553267; RRID:AB_394744
CD98-PE	Ebiosciences	Cat# 12-0981-82; RRID: AB_465793
CD62L FITC	BD Biosciences	Cat# 553150; RRID: AB_394665
CD44 APCeFluor 780	Ebiosciences	Cat# 47-0441-82; RRID:AB_1272244
B220 V500	BD Biosciences	Cat# 561227; RRID: AB_10562193
B220 PerCP-Cyanine5.5	Ebiosciences	Cat# 45-0452-82; RRID:AB_1107006
Thy 1.1 APC	Ebiosciences	Cat# 17-0900-82; RRID:AB_469420

**Chemicals, Peptides, and Recombinant Proteins**		

metformin hydrochloride	Sigma-Aldrich	D150959
phenformin hydrochloride	Sigma-Aldrich	P7045
rapamycin	Sigma-Aldrich	R0395
Proteinase K	Roche	3115836001
Diisononyl phthalate	Sigma-Aldrich	376663
Poly(dimethylsiloxane-*co*-methylphenylsiloxane)	Sigma-Aldrich	378488
Thiazolyl Blue Tetrazolium Bromide	Sigma-Aldrich	M5655

**Critical Commercial Assays**		

L-lactate assay kit	Abcam	Ab65331

### Contact for Reagent and Resource Sharing

Further information and requests for resources and reagents should be directed to and will be fulfilled by the Lead Contact, Grahame Hardie (d.g.hardie@dundee.ac.uk).

### Experimental Model and Subject Details

#### Mouse lines

Mice with *LoxP* sites flanking exon 5 of the *Pten* gene (Hagenbeek et al., 2004) were a gift from Hergen Spits. Mice with *LoxP* sites flanking exons 4 and 5 of the *Prkaa1* gene (Boudaba et al., 2018) were described previously. Transgenic mice expressing Cre recombinase from the *Lck* promoter (Takahama et al., 1998) were described previously. All mice used had been backcrossed onto the C57BL/6J background for at least six generations. Both males and females were used; their ages are detailed in Figures. Mice were housed in individually ventilated cages maintained at 20-22°C with free access to food and water. All experiments with live animals were approved by the Ethical Review Committee of the University of Dundee in accordance with the UK Animal (Scientific Procedures) Act 1986.

#### Cell lines and primary cultures

Established PTEN null T-ALL cells (M100, F04, F15 (Hagenbeek et al., 2004)) were a gift from Hergen Spits. Primary T-ALL cells were isolated by mechanical disruption of thymus using a syringe plunger and filtering the homogenized tissue through a 70 μm cell strainer. Established cells and primary T-ALL cells were cultured in Iscove’s Modified Dulbecco’s Medium (IMDM) with 10% (v/v) FBS and 1% (v/v) penicillin/streptomycin.

### Method Details

#### Mouse genotyping

Genotyping: a small piece of ear was digested with proteinase K (1 mg/ml) in 28 mM NaCl, 55 mM Tris, 0.1% SDS for 3 hr at 55°C. Samples (2 μl) were used to perform PCR.

*Pten*^*fl/fl*^ *primers:* forward, 5′-GCCTTACCTAGTAAAGCAAC-3′, reverse: 5′-GGCAAAGAATCTTGGTGTTAC-3′, which generated products of 280 bp (*Pten*^fl^) or 230 base pairs (wild-type). *Prkaa1*^*fl/fl*^ *primers:* forward, 5′-TTCAGGAAGGTATTGCTGCCATTAGG-3′,

reverse: 5′-GGATATGCCCAACCTCTCACGG-3′, which generated products of 680 bp (*PRKAA1*^fl^) or 584 bp (wild-type)

#### Flow cytometry

Cells were staining with fluorochrome-conjugated antibodies to detect CD4, CD8, CD71, CD98, CD62L, CD44, B220 and/or Thy1. Antibodies were diluted 1:200 and 1 × 10^6^ cells were stained in 100 μl. Live cells were gated according to forward scatter and side scatter. Data were acquired on a FACS Verse machine (Becton Dickinson) and analyzed using FlowJo software.

#### Cell survival assays using flow cytometry

Cells were incubated for 24 hr at the indicated biguanide concentration, washed with phosphate-buffered saline (PBS), incubated for 5 min with 4′,6-diamidino-2-phenylindole (DAPI, 20 μg/ml) in PBS, and washed again with PBS. Samples were analyzed on a FACS Verse flow cytometer (Becton Dickinson); this instrument is equipped with a volume sensor and gives absolute counts for all samples analyzed. Cells were identified on the basis of forward angle light scatter (FSC) and side angle light scatter (SSC) and DAPI fluorescence measured by excitation at 405 nm and emission detected at 448 ± 45 nm. Cells were distinguished from debris on the basis of FSC and SSC, and were further designated as alive or dead based on the absence or presence of DAPI, respectively.

#### MTT assays for cell proliferation

At the end of treatment (48 hr), Thiazolyl Blue Tetrazolium Bromide was added to the medium at 0.5 μg/μL for at least 24 hr. Water-insoluble MTT formazan crystals were solubilized with acidified isopropanol and intensity measured colorimetrically at 570 nm.

#### Measurement of ADP:ATP ratios

The levels of ADP and ATP were measured by capillary electrophoresis as described previously (Hawley et al., 2010), and the results expressed as ADP:ATP ratios.

#### LC:MS assays for biguanides in tissues

Plasma (50 μl) was mixed with 45 μl of acetonitrile and 5 μl of 12.5 μM metformin-d6 or 6.25 μM 1-phenylbiguanide hydrochloride (internal standards). Plasma samples were centrifuged at 15,000 rpm for 10 min at 4°C. Freeze-clamped livers were ground to powder in liquid N_2_ using a mortar and pestle. Tissue was dissolved at 50 μg/μl in acetonitrile. Sample (50 μl) was mixed with 45 μl of water plus 5 μl of 12.5 μM metformin-d6 or 6.25 μM of 1-phenylbiguanide hydrochloride. Liver samples were centrifuged at 15,000 rpm for 10 min at 4 degrees. After T cells were harvested, 20 μg were diluted into 20 μl of water plus 25 μl of acetonitrile and 5 μl of 12.5 μM metformin-d6 or 6.25 μM of 1-phenylbiguanide hydrochloride. Thymus samples were centrifuged at 15,000 rpm for 10 min at 4°C. Phenformin levels were measured using a TSQ Quantiva interfaced with an Ultimate 3000 Liquid Chromatography system (ThermoScientific), equipped with a C18 column (Atlantis T3, 50x1 mm, ID 3 μm (Part No: 186003713, Waters). Mobile phase buffer A consisted of 0.1% (v/v) formic acid and buffer B was 80% (v/v) acetonitrile. The column was maintained at 40°C and was equilibrated with 3% buffer B for 6 minutes at a constant flow rate of 0.06 mL/min. Aliquots of 1 μl of each sample were loaded onto the column and compounds eluted with a linear gradient to 10% buffer B within 2 min, then to 30% B within 5 min. Buffer B was then ramped up to 100% within 4 min and the column washed for a further 3 min. Eluents were sprayed into the TSQ Quantiva using ion Max NG ion source with ion transfer tube temperature set to 350°C and vaporizer temperature 125°C. The TSQ Quantiva was run in positive mode with a spray voltage of 2700, sheath gas 40 and Aux gas 10. Phenformin and internal standard (1-phenylbiguanide hydrochloride) were measured using multiple reaction monitoring mode (MRM) with optimized collision energies and radio frequencies previously determined by infusing pure compounds. Phenformin was measured using transitions (206.22 > 328.03 and 206.22 > 60.2) and internal standard was measured using transitions (178.44 > 60.8 and 178.44 > 119.19). A standard curve was prepared using phenformin with amounts ranging from 45 pM to 450 nM with the internal standard spiked in at a constant amount of 312 pM. The standard curve was run prior to running the samples using the same conditions, and was used to calculate the amount of phenformin in the samples. Three blanks were run between each sample to eliminate carryover. The same LC-MS system was used to detect and quantify metformin. LC conditions were optimized using a HILIC column (Atlantis HILIC Silica T3, 50 × 1 mm, ID 3 μm (Part No. 186002003, Waters). Mobile phase buffer A consisted of 0.1% (v/v) formic acid and buffer B was 2.5 mM ammonium formate in 95% (v/v) acetonitrile. The column was maintained at 32°C and was equilibrated with 100% buffer B for 5 min at a constant flow rate of 0.06 mL/min. Aliquots of 1 μL of each sample were loaded onto the column and an isocratic flow of 100% buffer B was applied for 4 min. Elution of compounds was achieved by a linear gradient from 100% B to 10%B within 5 min. Eluents were sprayed into the TSQ Quantiva using ion Max NG as described for phenformin. The TSQ Quantiva was run in positive mode with a spray voltage of 2700, sheath gas 35 and Aux gas 10. Metformin and deuterated metformin internal standard were measured using multiple reaction monitoring mode (MRM) with optimized collision energies and radio frequencies previously determined by infusing pure compounds. Two transitions (130.08 > 60.17 and 130.08 > 71.14) were used to monitor metformin and three (136.21 > 60.17, 136.21 > 77.17, 136.21 > 85.1) to detect deuterated metformin. A standard curve was generated with metformin ranging from 45 nM to 450 μM, and the internal standard spiked in at a constant amount of 232 pM. Three blanks were run between each sample to eliminate carryover.

#### Measurements of cellular oxygen consumption

Cellular oxygen consumption rate was measured using a Seahorse XF24 Extracellular Flux Analyzer according to manufacturers’ instructions, as specified previously (Hawley et al., 2010).

#### Measurements of nutrient uptake by cells

Nutrient uptake was carried out using 1 × 10^6^ cells resuspended in 0.4 mL of the medium specified below, layered over oil (di-isononyl phthalate and poly(dimethylsiloxane-*co*-methylphenylsiloxane, 1:1). After uptake for 4 min, cells were centrifuged to remove the medium, oil was aspirated and cell pellets resuspended in 1 mM NaOH. Radioactivity was measured using a scintillation counter. Glucose uptake was measured individually in glucose-free DMEM (Life Technologies) containing the glucose analog [^3^H]-2-deoxyglucose (1 μCi ml^−1^). Glutamine or leucine uptake was measured in HBSS with Ca^2+^ and Mg^2+^ (Life Technologies) containing [^3^H]-L-glutamine (1 μCi ml^−1^) or [^3^H]-L-leucine (1 μCi ml^−1^).

#### Lactate Assay

L-Lactate was measured with a colorimetric kit from Abcam (ab65331) following manufacturer’s instructions.

#### Other analytical procedures

SDS-PAGE was performed using precast Bis-Tris 4%–12% gradient polyacrylamide gels in the MOPS buffer system (ThermoFisher Scientific). Proteins were transferred to nitrocellulose membranes using the iBlot 2 system (ThermoFisher Scientific). Membranes were blocked for 1 hr in LICOR Odyssey blocking buffer. The membranes were probed with appropriate antibody (0.1–1 μg/ml) in TBS-Tween and 2% (w/v) non-fat dried skimmed milk except where the blotting enhancement system was used (as per manufacturers’ instructions). Detection was performed using secondary antibody (1 μg/ml) coupled to IR 680 or IR 800 dye, and the membranes scanned using the LICOR Odyssey IR imager. Protein concentrations were determined by Coomassie Blue binding with bovine serum albumin as standard (Bradford, 1976).

### Quantification and Statistical Analysis

#### Survival curves and other curve fitting

*In vivo* survival curves were plotted using using GraphPad Prism 6 for Mac OSX. *In vitro* cell survival data (Figures 3A and 3B) were fitted to the equation: Y = 100-(100^∗^X/(IC_50_+X)) by non-linear regression using GraphPad Prism 6 for Mac OSX.

#### Statistical analysis

Numbers of replicates and statistical significance is indicated in Figures or Figure legends. Numbers of replicates (n) refer to biological replicates, i.e., the number of mice or independent cell cultures analyzed. Significances of differences were estimated with GraphPad Prism 6 for Mac OSX, using 1-way or 2-way ANOVA as appropriate, and (unless stated otherwise) Sidak’s multiple comparison test. Significant differences are indicated either by asterisks: ^∗^p < 0.05, ^∗∗^p < 0.01, ^∗∗∗^p < 0.001, ^∗∗∗∗^p < 0.0001, or by daggers: †p < 0.05, ††p < 0.01, †††p < 0.001, ††††p < 0.0001.

## Supplemental Information

Document S1. Figures S1–S3

Document S2. Article plus Supplemental Information

## References

[bib1] Alessi D.R., Sakamoto K., Bayascas J.R. (2006). LKB1-dependent signaling pathways. Annu. Rev. Biochem..

[bib2] Algire C., Amrein L., Bazile M., David S., Zakikhani M., Pollak M. (2011). Diet and tumor LKB1 expression interact to determine sensitivity to anti-neoplastic effects of metformin in vivo. Oncogene.

[bib3] Boudaba N., Marion A., Huet C., Pierre R., Viollet B., Foretz M. (2018). AMPK re-activation suppresses hepatic steatosis but its downregulation does not promote fatty liver development. EBioMedicine.

[bib4] Bradford M.M. (1976). A rapid and sensitive method for the quantitation of microgram quantities of protein utilizing the principle of protein-dye binding. Anal. Biochem..

[bib5] Bridges H.R., Jones A.J., Pollak M.N., Hirst J. (2014). Effects of metformin and other biguanides on oxidative phosphorylation in mitochondria. Biochem. J..

[bib6] Chandel N.S., Avizonis D., Reczek C.R., Weinberg S.E., Menz S., Neuhaus R., Christian S., Haegebarth A., Algire C., Pollak M. (2016). Are metformin doses used in murine cancer models clinically relevant?. Cell Metab..

[bib7] Crofford O.B. (1995). Metformin. N. Engl. J. Med..

[bib8] El-Mir M.Y., Nogueira V., Fontaine E., Avéret N., Rigoulet M., Leverve X. (2000). Dimethylbiguanide inhibits cell respiration via an indirect effect targeted on the respiratory chain complex I. J. Biol. Chem..

[bib9] Evans J.M., Donnelly L.A., Emslie-Smith A.M., Alessi D.R., Morris A.D. (2005). Metformin and reduced risk of cancer in diabetic patients. BMJ.

[bib10] Faubert B., Boily G., Izreig S., Griss T., Samborska B., Dong Z., Dupuy F., Chambers C., Fuerth B.J., Viollet B. (2013). AMPK is a negative regulator of the Warburg effect and suppresses tumor growth in vivo. Cell Metab..

[bib11] Finlay D.K., Rosenzweig E., Sinclair L.V., Feijoo-Carnero C., Hukelmann J.L., Rolf J., Panteleyev A.A., Okkenhaug K., Cantrell D.A. (2012). PDK1 regulation of mTOR and hypoxia-inducible factor 1 integrate metabolism and migration of CD8+ T cells. J. Exp. Med..

[bib12] Foretz M., Hébrard S., Leclerc J., Zarrinpashneh E., Soty M., Mithieux G., Sakamoto K., Andreelli F., Viollet B. (2010). Metformin inhibits hepatic gluconeogenesis in mice independently of the LKB1/AMPK pathway via a decrease in hepatic energy state. J. Clin. Invest..

[bib13] Fullerton M.D., Galic S., Marcinko K., Sikkema S., Pulinilkunnil T., Chen Z.P., O’Neill H.M., Ford R.J., Palanivel R., O’Brien M. (2013). Single phosphorylation sites in Acc1 and Acc2 regulate lipid homeostasis and the insulin-sensitizing effects of metformin. Nat. Med..

[bib14] Grzes K.M., Swamy M., Hukelmann J.L., Emslie E., Sinclair L.V., Cantrell D.A. (2017). Control of amino acid transport coordinates metabolic reprogramming in T-cell malignancy. Leukemia.

[bib15] Hagenbeek T.J., Spits H. (2008). T-cell lymphomas in T-cell-specific PTEN-deficient mice originate in the thymus. Leukemia.

[bib16] Hagenbeek T.J., Naspetti M., Malergue F., Garçon F., Nunès J.A., Cleutjens K.B., Trapman J., Krimpenfort P., Spits H. (2004). The loss of PTEN allows TCR αβ lineage thymocytes to bypass IL-7 and Pre-TCR-mediated signaling. J. Exp. Med..

[bib17] Hawley S.A., Ross F.A., Chevtzoff C., Green K.A., Evans A., Fogarty S., Towler M.C., Brown L.J., Ogunbayo O.A., Evans A.M., Hardie D.G. (2010). Use of cells expressing γ subunit variants to identify diverse mechanisms of AMPK activation. Cell Metab..

[bib18] Jeon S.M., Chandel N.S., Hay N. (2012). AMPK regulates NADPH homeostasis to promote tumour cell survival during energy stress. Nature.

[bib19] Kishton R.J., Barnes C.E., Nichols A.G., Cohen S., Gerriets V.A., Siska P.J., Macintyre A.N., Goraksha-Hicks P., de Cubas A.A., Liu T. (2016). AMPK is essential to balance glycolysis and mitochondrial metabolism to control T-ALL cell stress and survival. Cell Metab..

[bib20] Kordes S., Pollak M.N., Zwinderman A.H., Mathôt R.A., Weterman M.J., Beeker A., Punt C.J., Richel D.J., Wilmink J.W. (2015). Metformin in patients with advanced pancreatic cancer: a double-blind, randomised, placebo-controlled phase 2 trial. Lancet Oncol..

[bib21] Owen M.R., Doran E., Halestrap A.P. (2000). Evidence that metformin exerts its anti-diabetic effects through inhibition of complex 1 of the mitochondrial respiratory chain. Biochem. J..

[bib22] Rolf J., Zarrouk M., Finlay D.K., Foretz M., Viollet B., Cantrell D.A. (2013). AMPKα1: a glucose sensor that controls CD8 T-cell memory. Eur. J. Immunol..

[bib23] Ross F.A., MacKintosh C., Hardie D.G. (2016). AMP-activated protein kinase: a cellular energy sensor that comes in 12 flavours. FEBS J..

[bib24] Shackelford D.B., Abt E., Gerken L., Vasquez D.S., Seki A., Leblanc M., Wei L., Fishbein M.C., Czernin J., Mischel P.S., Shaw R.J. (2013). LKB1 inactivation dictates therapeutic response of non-small cell lung cancer to the metabolism drug phenformin. Cancer Cell.

[bib25] Suissa S., Azoulay L. (2014). Metformin and cancer: mounting evidence against an association. Diabetes Care.

[bib26] Takahama Y., Ohishi K., Tokoro Y., Sugawara T., Yoshimura Y., Okabe M., Kinoshita T., Takeda J. (1998). Functional competence of T cells in the absence of glycosylphosphatidylinositol-anchored proteins caused by T cell-specific disruption of the Pig-a gene. Eur. J. Immunol..

[bib27] Woods A., Salt I., Scott J., Hardie D.G., Carling D. (1996). The α1 and α2 isoforms of the AMP-activated protein kinase have similar activities in rat liver but exhibit differences in substrate specificity in vitro. FEBS Lett..

[bib28] Zhou G., Myers R., Li Y., Chen Y., Shen X., Fenyk-Melody J., Wu M., Ventre J., Doebber T., Fujii N. (2001). Role of AMP-activated protein kinase in mechanism of metformin action. J. Clin. Invest..

