# Phenformin, But Not Metformin, Delays Development of T Cell Acute Lymphoblastic Leukemia/Lymphoma via Cell-Autonomous AMPK Activation

**DOI:** 10.1016/j.celrep.2025.115956

**Published:** 2025-06-17

**Authors:** Diana Vara-Ciruelos, Madhumita Dandapani, Fiona M. Russell, Katarzyna M. Grzes, Abdelmadjid Atrih, Marc Foretz, Benoit Viollet, Douglas J. Lamont, Doreen A. Cantrell, D. Grahame Hardie

## Main text

(Cell Reports *27*, 690–698.e1–e4; April 16, 2019)

A reader pointed out a problem with Figure 4E in this paper. The anti-phospho-4EBP1 western blots for F04 and F15 cells were flagged as potential duplicates. When the original data was requested by the editor, the authors realized that one of the image files was accidentally mislabelled. Although the phospho-4EBP1 blots for M100 cells remain valid, it is not possible to say whether the duplicate blots came from F04 or F15 cells, and the third blot could not be located. However, even if the anti-phospho-4EBP1 blots are ignored, the conclusions drawn from Figure 4E are not affected because: (1) it is already well established that AMPK activators (e.g., phenformin) and rapamycin inhibit mTORC1 via distinct mechanisms that are well understood, and (2) inhibition of mTORC1 in these experiments was also monitored using a direct marker (S6KI phosphorylation) as well as indirect markers (RPS6 phosphorylation and expression of HIF-1α, ALDOA and LDHA). The authors apologize for their mistake and assert that the 4EBP1 phosphorylation panel in Figure 4E is not necessary to support the overall conclusions of the paper.

