# Supplementary material for: Phenformin, But Not Metformin, Delays Development of T Cell Acute Lymphoblastic Leukemia/Lymphoma via Cell-Autonomous AMPK Activation
Source: Cell Rep. 2019 Apr 16;27(3):690–698.e4. doi: 10.1016/j.celrep.2019.03.067 (PMC6484776; doi:10.1016/j.celrep.2019.03.067)
Supplement: Document S1. Figures S1–S3 [file mmc1.pdf]

Cell Reports, Volume *27*

Supplemental Information

Phenformin, But Not Metformin, Delays Development of T Cell Acute Lymphoblastic Leukemia/Lymphoma via Cell-Autonomous AMPK Activation

Diana Vara-Ciruelos, Madhumita Dandapani, Fiona M. Russell, Katarzyna M. Grzes, Abdelmadjid Atrih, Marc Foretz, Benoit Viollet, Douglas J. Lamont, Doreen A. Cantrell, and D. Grahame Hardie

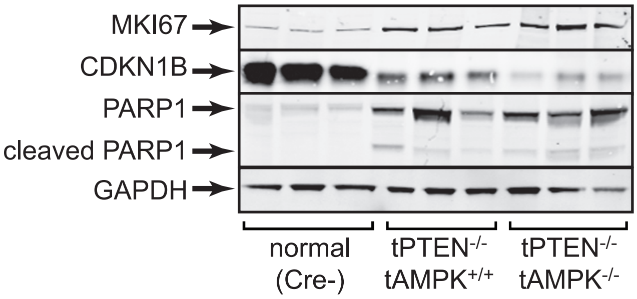

Figure S1: Markers of proliferation/apoptosis in normal thymus or lymphomas of different genotypes (n = 3). Quantification of blots from a larger number of samples is shown in Fig. 1E.

Vara-Ciruelos et al Supplementary Figure S2 (related to Fig. 1)

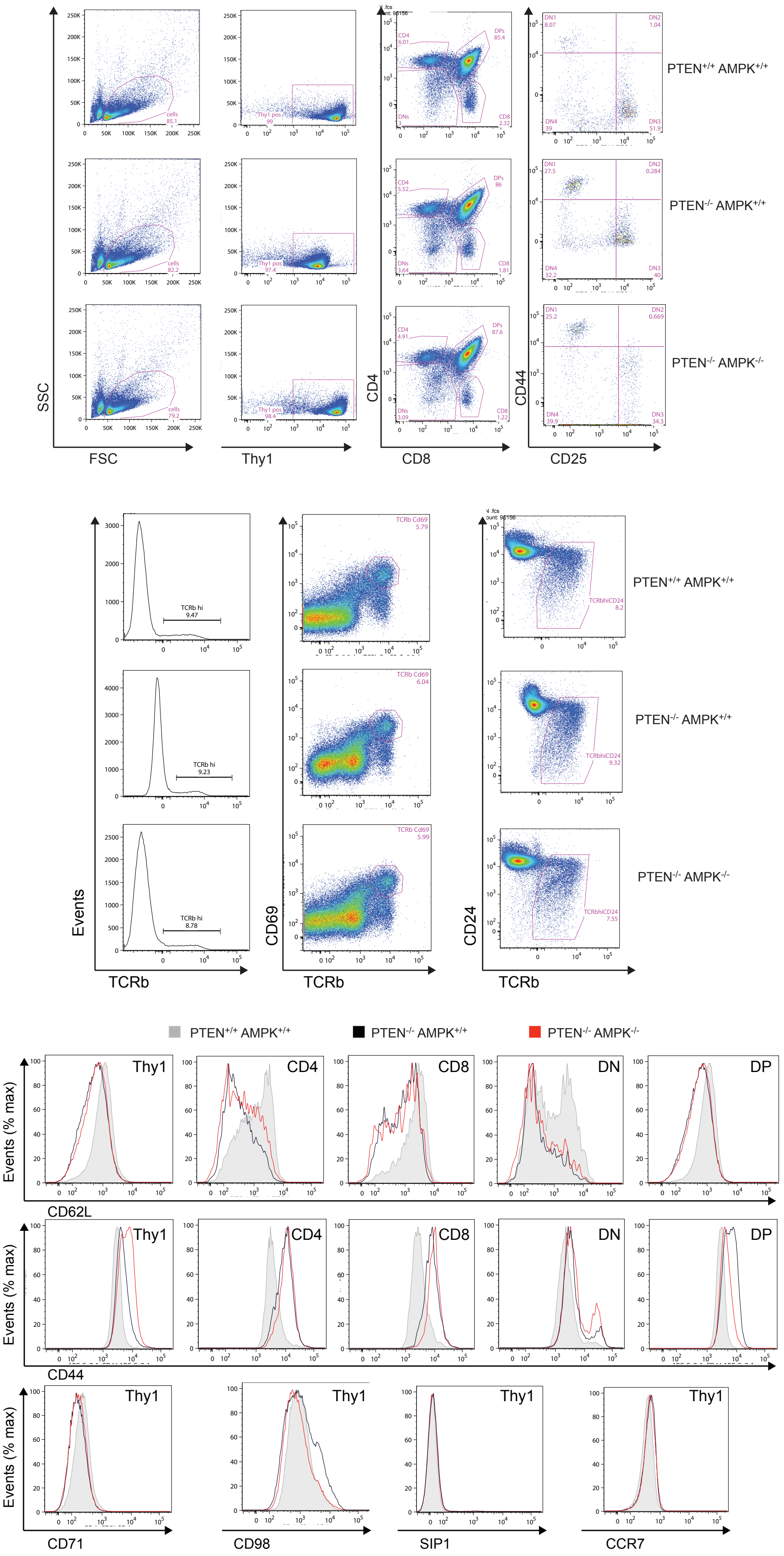

Figure S2: Expression of T cell markers is very similar in thymocytes from PTEN knockouts and PTEN/AMPK double knockouts

Vara-Ciruelos et al Supplementary Figure 3 (related to Fig. 2)

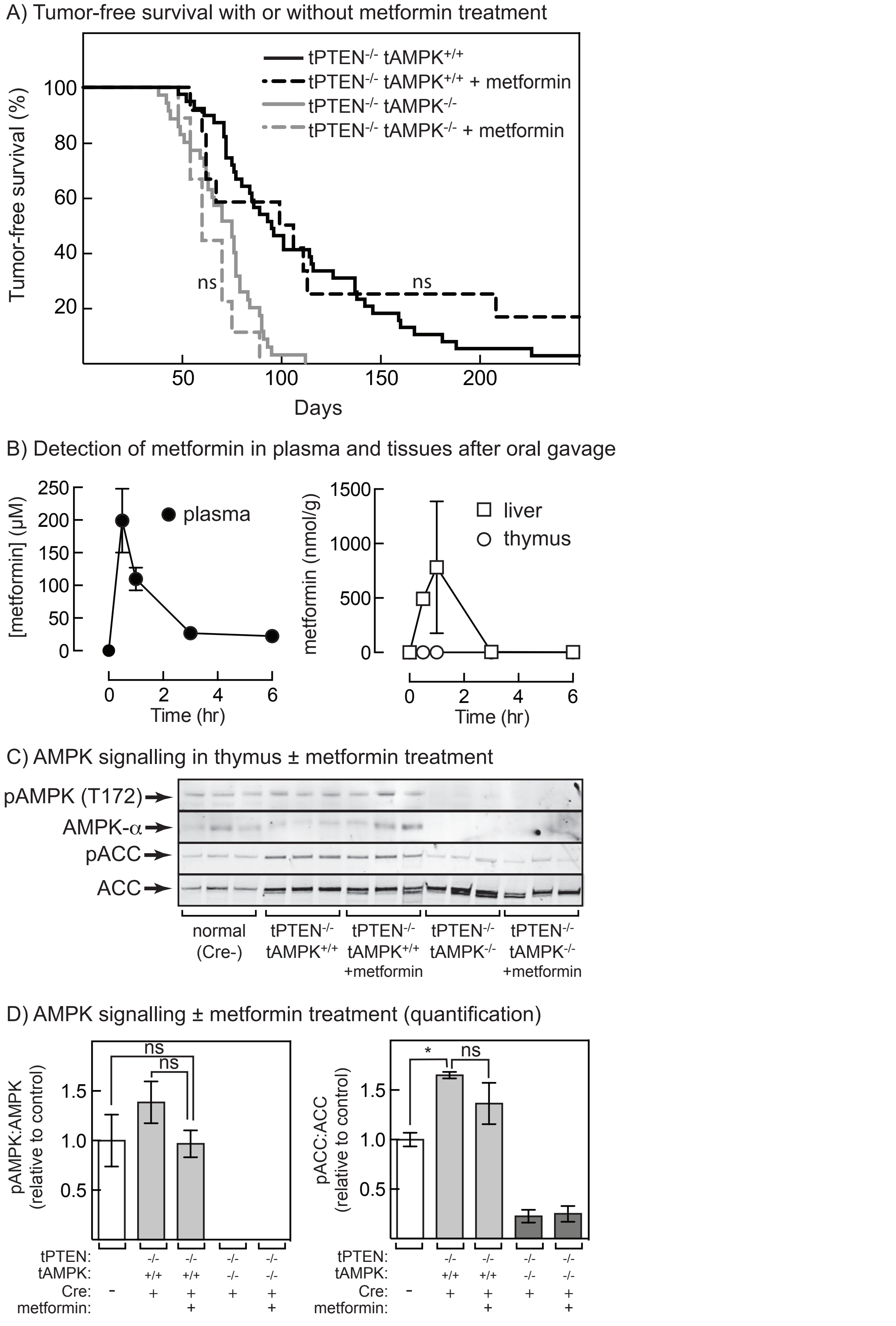

Figure S3: Effect of metformin treatment in vivo on tumor-free survival and cell signalling in thymus. (A) Effect of administration of metformin in drinking water (400 mg/kg) on tumor-free survival in tPTEN$^{-/-}$ tAMPK$^{+/+}$ or tPTEN$^{-/-}$ tAMPK$^{-/-}$ mice. The data for untreated controls are as in Fig. 1A. The effects of metformin treatment were not significant (Log-rank, Mantel Cox) in either genotype. (B) Effect of time after oral gavage with metformin on its recovery (estimated by LC:MS) in plasma (left) and liver and thymus (right) in wild type mice (29-42 days of age). Results are mean ± SEM (n = 3). (C) Expression and phosphorylation of AMPK and ACC in thymus or thymic lymphomas of mice treated with and without metformin (three representative mice for each genotype or treatment). (D) Quantification of Western blotting data for all mice analyzed; results are mean ± SEM (n = 3). Results significantly different by 1-way ANOVA are indicated by asterisks; ns, not significant.
